# Relationship of nutritional behaviors and parent–child interactions with developmental domains of Iranian toddlers: a cross-sectional study

**DOI:** 10.1186/s12887-024-04948-z

**Published:** 2024-07-31

**Authors:** Parastoo Faghani, Nasrin Nikpeyma, Shima Haghani, Zahra Amrollah Majdabadi, Shahzad Pashaeypoor

**Affiliations:** 1 MSc of Nursing, Department of Community Health and Geriatric Nursing, School of Nursing and Midwifery, Tehran University of Medical Sciences, Tehran, Iran; 2 PhD of Nursing, Department of Community Health and Geriatrics Nursing, School of Nursing and Midwifery, Tehran University of Medical Sciences, Tehran, Iran; 3 MSc in Biostatistics Nursing Care Research Center, Iran University of Medical Sciences, Tehran, Iran

**Keywords:** Development, Parent-child relationship, Nutrition, Toddler

## Abstract

**Background:**

Health service providers closely monitor the developmental state of toddlers to identify the factors affecting this process because any defect during this period will cause irreversible damage. Therefore, this study investigated the relationship of nutritional behaviors and parent–child interactions with the developmental domains of Iranian toddlers.

**Methods:**

This cross-sectional, descriptive-analytical study was conducted on 341 toddlers aged 12–36 months covered by comprehensive health centers in the south of Tehran in 2021–2022. The participants were selected through single-stage cluster sampling. To this end, 16 comprehensive health centers were randomly selected, and then some of the clients from each center were randomly selected as the sample. The required data were collected through the Ages and Stages Questionnaire (ASQ), the Children’s Eating Behavior Questionnaire (CEBQ), the Child-Parent Relationship Scale (CPRS), and a demographics form. They were then analyzed statistically using descriptive and inferential statistics in SPSS-21, considering a significance level of *p* < 0.05.

**Results:**

The results showed that most participants were normal in all developmental domains (communication, gross motor, fine motor, personal-social, and problem-solving), with a mean developmental delay ranging from 1.8 to 7%. The most serious problem of participants requiring medical referral was related to gross motor (7%) with a mean of 54.35 ± 7.28 followed by communication (6.5%) with a mean of 49.41 ± 9.67. The mean nutritional behavior of participants was 77.9 ± 21.7. A significant relationship was found between the nutritional behaviors of participants and the problem-solving domain of development (*p* = 0.018). The results also indicated a mean parent-child interaction score of 94.26 ± 12.63. There was a significant relationship between parent-child interactions and the communication area of development (*p* = 0.04).

**Conclusion:**

Since some areas of toddler development are influenced by children’s nutritional behavior and parent-child interactions, it is necessary to train families to identify, monitor, and correct the factors affecting the development of their children. Health system officials and planners are also recommended to develop interventions to improve the nutritional behaviors of children and parent-child interactions.

## Background

Achieving full growth and development is one of the most important elements of children’s rights because children are regarded as the most valuable human capital and vulnerable age group [1]. The toddler stage is one of the most crucial in child development as it is considered an essential, unique, and irreversible phase in children’s development [2]. Childhood is the foundation for adult growth and development, and most physical and mental disorders in adulthood result from neglecting childhood problems and failing to properly guide children through the course of development [3]. Development refers to changes in a child’s thinking and behavior over time caused by genetic, environmental, psychological, and social factors. These changes occur in a specific order, are well-organized, and to some extent predictable [4]. Toddlers develop in communication, motor, cognitive, and personal-social domains [2]. A delay in any developmental area indicates the absence of developmental characteristics according to the child’s age [5]. Approximately 200 million children worldwide have a poor developmental status. Evidence suggests that the prevalence of developmental disorders is higher in developing countries than in other parts of the world, and this rate has been estimated to range from 18 to 22% in Iran [6]. Despite the unknown main cause of developmental delay, evidence suggests that certain factors, such as parent-child relationships, family conflicts, child neglect, lack of warm parental relationships, and insecure attachment styles, increase the risk of behavioral problems and developmental disorders in children [7–9]. Studies have also shown that nutrition significantly influences children’s physical and cognitive development. Children who experience poverty or poor nutrition have lower IQs, weaker cognitive abilities, and more behavioral problems [10–12]. Therefore, correct parent-child interactions and healthy nutrition seem to be crucial factors affecting children’s ability to grow and develop normally. Parent-child relationships significantly influence children’s social, emotional, and cognitive development. In addition, proper nutrition affects children’s physical and mental development. It is, hence, necessary to pay special attention to the development of toddlers and the factors affecting this process because any defect during this period will irreversibly damage families and the whole society [10]. Moreover, children’s developmental disorders can impose heavy financial burdens on the family and society [12].

Monitoring children’s development is one of the responsibilities of health service providers, who also play a major role in preventing and diagnosing developmental disorders. They can instruct parents, assist them in becoming familiar with their responsibilities during this period, and prevent many developmental disorders by understanding toddler development principles and the factors influencing this process [13]. Parents also have an essential role in this regard because children will develop more effectively if they receive parental care of higher quality, particularly in the first three years of life. As a result, parents’ understanding of child care plays a significant role in the child’s overall development [4]. To intervene therapeutically and prevent irreversible complications, it is therefore essential to identify the factors influencing children’s developmental disorders. Early diagnosis also facilitates early treatment, improving children’s developmental disorders and health [11]. Considering the importance of children’s development and the factors affecting this process, the lack of reliable evidence in this field, and the cultural, social, and economic differences between different societies, this study investigates the relationship between nutritional behaviors and parent-child interactions with developmental domains of Iranian toddlers.

## Methods

### Design and sample

This cross-sectional, descriptive-analytical study was conducted on 341 toddlers aged 1 to 3 years covered by comprehensive health centers in Tehran in 2021–2022. The inclusion criteria were no obvious congenital abnormalities, no history of hospitalization for postnatal non-obstetric reasons (including accidents and trauma), and no cognitive and psychological disorders (self-reported by mothers). The exclusion criteria were child hospitalization, incomplete questionnaires, and unwillingness of parents to continue the study.

After inserting into the following formula, the minimum required sample size of 340 was obtained to estimate the developmental frequency of toddlers at the 95% confidence level and with an estimation accuracy of d = 0.05 (it should be noted that Dare et al. estimated a developmental status of 67%) [14], and considering a 5% attrition rate (17 individuals), finally 357 toddlers were included in the study.

Participants were selected through cluster sampling. To this end, a list of Tehran’s comprehensive health centers was prepared, and 16 centers were chosen randomly from a pool of 95. In this manner, the names of these centers were written on 95 different cards in no particular order and placed in a box. The cards were mixed, and 16 cards were selected, one after the other. The author visited the selected centers and used the SIB system to randomly select two toddlers (a total of 22 toddlers from each center) who met the inclusion criteria from among the 11 age groups (12 to 36 months). Of 357 participants, 16 were excluded from the study because they did not apply, were later found not to meet the inclusion criteria, had provided false information, or were unwilling to cooperate with the author. Finally, the study was conducted on 341 participants.

$$\:n=\frac{{z}_{1-\raisebox{1ex}{$\propto\:$}\!\left/\:\!\raisebox{-1ex}{$2$}\right.}^{2}\:pq}{{d}^{2}}=\frac{{1.96}^{2}\times\:0.67\times\:0.33}{{0.05}^{2}}=340$$

### Data collection

The required data were collected through the ages and stages questionnaire (ASQ), the children’s eating behavior questionnaire (CEBQ), the child-parent relationship scale (CPRS), and a demographics form.

#### The demographics form

This author-made questionnaire consisted of items about gender, birth rank, child’s age, and child’s birth weight.

**Ages and stages questionnaire (ASQ)**: The developmental status of participants was measured using ASQ. Developed by Bricker and Squires (1999) [15] and translated and standardized in Farsi by Sajjedi et al. [16], this tool consists of 19 tests for toddlers aged 4, 6, 8, 10, 12, 14, 16, 18, 20, 22, 24, 27, 30, 33, 36, 42, 48, 54, and 60 months old. There are 30 items for each age group, with 6 items covering the five developmental domains of communication, gross motor, fine motor, problem-solving, and personal-social skills. Each item has three options: “No” when the child has never done that activity (0 points), “Yes” when the child can do that activity (10 points), and “Sometimes” when the child does that activity sometimes (5 points). The developmental status of participants was assessed by comparing the total scores on each domain with the predetermined cut-off points in the test’s instructions. Suppose the child does not meet the corresponding cut-off point in the five domains. In that case, there is a problem in that area, and the necessary specialized measures should be taken for the children to ensure their health or diagnose possible disorders and diseases. This study’s cut-off point for developmental disorder was one unit of standard deviation below the mean. If the child’s score in each domain is equal to or greater than the cut-off point, there is no problem; if it is 1 to 2 units of standard deviation less than the cut-off point, the child should be monitored in that domain, and if it is 2 units of standard deviation equal to or less than the cut-off point, the child should be referred for follow-up and accurate evaluations [16]. Sajjedi et al. [16] standardized and validated ASQ. The questionnaires’ construct validity was verified using the factor analysis method, and a Cronbach’s alpha of 0.79 was calculated. This tool was also standardized in Iran by the University of Welfare and Rehabilitation Sciences, the Ministry of Health and Medical Education, and other related organizations from 2011 to 2016. Its reliability, validity, and ability to identify developmental disorders were reported to be 0.94, 0.84, and more than 96%, respectively [17]. In this study the Cronbach’s α values of the present questionnaire were found to be at acceptable levels for total score (0.84).

**Children’s eating behavior questionnaire (CEBQ)**: This questionnaire was developed by Wardle et al. (2001) in England [18]. It was translated into Farsi, and its validity was assessed in Iran by Nasirzadeh et al. (2017); KMO for this questionnaire was reported as 0.83, and internal consistency of the whole questionnaire was 0.74 through Cronbach’s alpha and 0.71 through the test-retest method [19]. Also In this study the Cronbach’s α values of the present questionnaire were found to be at acceptable levels for total score (0.973). This questionnaire consists of 35 items scored on a 5-point Likert scale (from 0: never to 4: always). Every 3 to 6 items correspond to one of the subscales of this questionnaire: food responsiveness (FR), enjoyment of food (EF), emotional overeating (EOE), desire to drink (DD), satiety responsiveness (SR), slowness in eating (SE), emotional undereating (EUE), and food fussiness (FF). The first four subscales on this questionnaire are considered scales of food tendency, indicating a positive desire to eat. In contrast, the remaining four subscales are considered scales of food avoidance, indicating a negative tendency towards food. Higher scores on this questionnaire indicate more nutritional problems.

**Child-parent relationship scale (CPRS)**: Developed by Pianta in 2011 in France, CPRS consists of 33 items that measure parents’ perception of their relationship with their child [20]. The subscales of this tool include conflict (17 items), intimacy (10 items), dependence (6 items), and overall positive relationship (sum of all domains). The items of this self-report questionnaire are scored on a 5-point Likert scale (from 5: true to 1: not true). In this scale, the conflict and dependence subscale scores are reversed to obtain the overall score; higher scores indicate a positive relationship between parents and children [20]. This questionnaire has been translated in Iran, and its content validity and reliability have been confirmed in numerous studies. The Cronbach’s alpha for the whole scale was reported to range between − 0.88 and 0.86, 0.82 and 0.85 for intimacy and dependence, and 0.78 and 0.85 for conflict [21]. In the above study, the Cronbach’s alpha was within the normal and acceptable range (0.816).

After obtaining the necessary permits, the author visited the comprehensive health centers in Tehran to select the research participants from those who met the inclusion criteria. The author briefed the participants on the research objectives and procedure, assured them that their personal information would be kept confidential, and emphasized the safe procedure of the research. Then, the author completed the questionnaires for each participant in an interview. The obtained data were analyzed statistically using descriptive statistics (mean and standard deviation) and inferential statistics (Pearson correlation coefficient, chi-square test, Mann-Whitney U test, ANOVA, and Kruskal-Wallis test) in SPSS-21.

## Findings

Demographic data showed that 178 participants (51.2%) were boys, and 163 (47.6%) were girls. Participants’ mean age and birthweight were 22.9 ± 7.52 months and 3.53 ± 0.55 kg, respectively (Table 1).

**Table 1:** Frequency distribution of demographic characteristics of participants

Demographic characteristics		Frequency	Percentage
Gender	Female	163	47.8
	Male	178	52.2
Birth rank	First	102	29.9
	Second	124	36.4
	Third	99	29
	Fourth	16	4.7
Age (month)	12–16	93	27.3
	18–24	124	36.4
	Over 24	124	36.4
	Mean ± Standard deviation Minimum-Maximum	22.5 ± 7.52 12–36	
Birthweight (kg)	Underweight (below 2.5)	15	4.4
	Normal (2.5-4)	320	93.8
	Overweight (over 4)	6	1.8
	Mean ± Standard deviation Minimum-Maximum	3.53 ± 0.55 2–5	

The results also showed that most participants were normal in all developmental domains, with a mean developmental delay ranging from 1.8 to 7%. The most serious problem of participants requiring medical referral was related to gross motor (7%) with a mean of 54.35 ± 7.28 followed by communication (6.5%) with a mean of 49.41 ± 9.67. In addition, the least serious problem requiring medical referral was related to fine motor (1.8%) with a mean of 53.4 ± 7.39 (Table 2).

**Table 2:** Frequency distribution of developmental status of participants

Developmental domains	Normal		Requiting re-monitoring		Requiring medical referral		mean ± standard deviation
	frequency	percentage	frequency	percentage	frequency	percentage	
**Communication**	247	72.4	72	21.1	22	6.5	49.41 ± 9.67 10–60
**Gross motor**	278	81.5	39	11.4	24	7	54.35 ± 7.28 15–60
**Fine motor**	319	93.5	16	4.7	6	1.8	53.43 ± 7.39 20–60
**Problem-solving**	232	68	89	26.1	20	5.9	49.72 ± 7.26 20–60
**Personal-social**	288	84.5	39	11.4	14	4.1	52.98 ± 8.4 10–60

As shown in Table 3, the mean nutritional behavior of participants was 77.921.7, which was close to the tool’s mean score of 70. Scores were then calculated on a scale of 0 to 100 to compare the nutritional behavior dimensions. The results showed that food avoidance, with a mean of 56.8 ± 15.2, was more frequent than food tendency, with a mean of 54.2 ± 16.2. Slowness in eating had the lowest mean score (59.03 ± 17.3), and satiety responsiveness had the highest mean score (61.3 ± 17.3) in the food avoidance dimension. Food responsiveness and enjoyment of food obtained the lowest (47 ± 17) and highest (58 ± 17.1) mean scores, respectively, in the food tendency dimension.

**Table 3:** Mean and standard deviation of nutritional behavior of participants

Nutritional behavior		Mean	Standard deviation	Based on 0 to 100	
				Mean	Standard deviation
Food tendency	Food responsiveness (0–20)	9.4	3.4	47.03	17.04
	Enjoyment of food (0–20)	11.6	3.42	58	17.1
	Emotional overeating (0–12)	6.83	2.25	56.94	18.77
	Desire to drink (0–12)	6.85	2.24	57.08	18.73
	Total food tendency score (0–64)	34.7	10.4	54.2	16.23
Food avoidance	Satiety responsiveness (0–16)	9.8	2.76	61.27	17.35
	Slowness in eating (0–16)	9.44	2.77	59.03	17.35
	Food fussiness (0–24)	14.17	3.93	59.04	16.4
	Emotional undereating (0–16)	9.77	2.77	61.08	17.35
	Total food avoidant score (0–76)	43.2	11.59	56.83	15.25
Nutritional behavior (0-140)		77.88	21.66	55.63	16.47

The study results indicated that “problem-solving” was the only developmental domain showing a significant relationship with the nutritional behavior of participants (*p* = 0.018). According to Tukey’s multiple comparison tests, the mean score of nutritional behavior with normal problem-solving (84.15 ± 22.8) was significantly higher than that of participants requiring medical referral (69.9 ± 20.8) (Table 4).

**Table 4:** Relationship between developmental domains and nutritional behavior of participants

Nutritional behavior Developmental domains		Food tendency		Food avoidance		Nutritional behavior	
		Mean	Standard deviation	Mean	Standard deviation	Mean	Standard deviation
**Communication**	**Requiring medical referral**	34.09	13.89	44.77	12.05	78.86	23.69
	**Requiting re-monitoring**	35.36	11.55	45.88	13.64	81.25	23.91
	**Normal**	37.36	11.46	45.75	12.76	83.11	22.06
	**Test result**	F = 1.428, *P* = 0.241		F = 0.066, *P* = 0.935		F = 0.488, *P* = 0.615	
**Gross motor**	**Requiring medical referral**	37.1	12.84	50.08	13.58	87.2	23.01
	**Requiting re-monitoring**	38.53	10.25	46.66	13.93	85.2	22.14
	**Normal**	36.44	11.75	45.2	12.62	81.65	22.54
	**Test result**	F = 0.566, *P* = 0.568		F = 1.711, *P* = 0.182		F = 1.001, *P* = 0.369	
**Fine motor**	**Requiring medical referral**	27.33	9.62	40	17.44	67.33	23.48
	**Requiting re-monitoring**	37.43	12.33	43.18	13.63	80.62	24.61
	**Normal**	36.87	11.62	45.95	12.75	82.82	22.38
	**Test result**	F = 2.012, *P* = 0.135		F = 0.954, *P* = 0.386		F = 1.45, *P* = 0.236	
**Problem-solving**	**Requiring medical referral**	31.25	11.17	38.7	10.76	69.95	20.82
	**Requiting re-monitoring**	35.69	10.77	45.11	12.53	80.8	21.24
	**Normal**	37.59	11.91	46.55	13.01	84.15	22.85
	**Test result**	F = 3.244, *P* = 0.04		F = 3.617, *P* = 0.028		F = 4.048, *P* = 0.018	
**Personal-social**	**Requiring medical referral**	30.85	11.68	42	12.12	72.85	20.57
	**Requiting re-monitoring**	35.53	12.57	45.35	12.81	80.89	23.57
	**Normal**	37.17	11.48	45.94	12.92	83.12	22.43
	**Test result**	F = 2.206, *P* = 0.112		F = 0.644, *P* = 0.526		F = 1.495, *P* = 0.226	

According to Table 5, the mean score of parent-child interactions was 94.26 ± 12.63, less than the tool’s average score of 99, suggesting less conflict between parents and their children. Higher mean scores imply less conflict since the conflict score is interpreted reversely. Scores were calculated on a scale of 0 to 100 to compare the dimensions of parent-child interactions. The results revealed that parent-child interactions were highest in the conflict dimension (55.46 ± 11.69) and lowest in the intimacy dimension (36.57 ± 11.26).

**Table 5:** Mean and standard deviation of dimensions of parent-child interactions of participants

Parent-child interaction			Based on 0 to 100	
	Mean	Standard deviation	Mean	Standard deviation
**Conflict (17–85)**	54.71	7.95	55.46	11.69
**Intimacy (50 − 10)**	24.64	3.5	36.57	11.26
**Dependence (30 − 6)**	14.61	2.9	37.14	12.09
**Parent-child interaction (165 − 33)**	94.26	12.63	46.41	9.56

The results demonstrated a significant relationship between communication domain and parent-child interaction (*p* = 0.042). The mean score of parent-child interaction in normal participants (95.1 ± 11.7) was significantly higher than in participants requiring medical referral (88.5 ± 17.4). A significant relationship was also found between the communication domain and the conflict dimension of parent-child interaction (*p* = 0.013). The pairwise comparison showed a significantly higher mean score of conflict in participants with normal communication (55.3 ± 7.22) than in those requiring medical referral (50.3 ± 10.68) (Table 6).

**Table 6:** Relationship between developmental domains and parent-child interaction dimensions in participants

Parent-child interaction Developmental domains		Conflict		Intimacy		Dependence		Parent-child interaction	
		Mean	Standard deviation	Mean	Standard deviation	Mean	Standard deviation	Mean	Standard deviation
**Communication**	**Requiring medical referral**	50.36	10.68	23.63	5.16	14.50	3.41	88.5	17.42
	**Requiting re-monitoring**	53.97	8.15	24.15	3.47	14.97	3.02	93.09	11.55
	**Normal**	55.32	7.22	24.85	4.47	14.93	2.82	95.11	11.77
	**Test result**	F = 4.42, *P* = 0.013		F = 1.257, *P* = 0.286		F = 0.244, *P* = 0.784		F = 3.203, *P* = 0.042	
**Gross motor**	**Requiring medical referral**	55.25	5.88	25.33	4.06	15.83	2.72	96.41	10.18
	**Requiting re-monitoring**	54.41	8.39	23.38	3.84	15.69	2.57	93.48	12.54
	**Normal**	54.71	8.06	24.74	4.61	14.72	2.93	94.18	12.85
	**Test result**	F = 0.083, *P* = 0.921		F = 1.881, *P* = 0.154		F = 3.227, *P* = 0.041		F = 0.426, *P* = 0.653	
**Fine motor**	**Requiring medical referral**	57.5	7.03	24.5	3.01	13.66	2.25	95.66	6.28
	**Requiting re-monitoring**	56.12	7.85	26.06	2.54	14.81	1.68	97.00	8.06
	**Normal**	54.59	7.79	24.56	4.59	14.94	2.96	94.10	12.90
	**Test result**	F = 0.654, *P* = 0.52		F = 0.847, *P* = 0.429		F = 0.579, *P* = 0.561		F = 0.438, *P* = 0.646	
**Problem-solving**	**Requiring medical referral**	56.25	6.24	25.45	4.09	15.2	2.76	96.90	10.82
	**Requiting re-monitoring**	54.95	7.53	24.38	4.10	15.13	2.97	94.47	11.56
	**Normal**	54.49	8.25	24.65	4.69	14.8	2.89	93.95	13.17
	**Test result**	F = 0.5, *P* = 0.607		F = 0.468, *P* = 0.627		F = 0.514, *P* = 0.599		F = 0.515, *P* = 0.598	
**Personal-social**	**Requiring medical referral**	53	9.41	24	5.53	15.28	3.33	92.28	15.75
	**Requiting re-monitoring**	52.97	10.18	23.74	5.40	14.74	2.94	91.46	16.18
	**Normal**	55.03	7.51	24.78	4.31	14.92	2.88	94.73	11.89
	**Test result**	F = 1.503, *P* = 0.244		F = 1.054, *P* = 0.35		F = 0.182, *P* = 0.834		F = 1.338, *P* = 0.264	

*Analysis of variance (ANOVA)

## Discussion

This study investigated the relationship of nutritional behaviors and parent-child interactions with the developmental domains of Iranian toddlers living south of Tehran. The results showed that most participants were normal in all developmental domains, with a mean developmental delay ranging from 1.8 to 7%. Consistent with this result, most previous studies found that most children were developing within the normal range [2] and that the prevalence of developmental delay in toddlers was 6.4% [22]. In Iran, the results of a study showed that 67.5% of the examined children had a normal developmental process, while 6.1% of them were in the areas of abnormal development [14]. Also, in the study of Sajedi et al., developmental delay between 3.69% and 4.31% was reported in different areas [16].

The most serious problem of participants requiring medical referral was related to gross motor (7%), followed by communication (6.5%). Valla et al. (2015) discovered that most children aged 4 to 12 months had a developmental delay in gross motor [23]. Another study found that children’s communication skills were the least developed, while problem-solving skills were the most developed [24]. These results are consistent with our findings. In contrast, other evidence and data show that 18.8% of children have developmental delays, with communication problems accounting for 1.7% of those cases and gross motor skill problems accounting for the fewest delays (0.9%) [6]. It is critical to pay attention to the developmental domains of toddlers, particularly communication and gross motor because they can serve as the foundation for the children’s communication, cognitive, motor, language, and emotional-social abilities and significantly affect their development [5].

The results on toddlers’ nutritional behavior revealed a higher mean score of food avoidance than food tendency. Other studies have identified inappropriate eating behaviors in toddlers, such as food avoidance, slowness in eating, and satiety responsiveness [13, 25], consistent with our findings. Leuba et al. (2022) also reported inadequate nutritional behaviors in toddlers as eating less, removing some nutrients from staple foods, avoiding eating, and fighting while eating [26]. The family’s eating habits play a significant role in developing toddlers’ eating habits, and mothers’ awareness of the nutritional needs of toddlers can significantly enhance those behaviors [25]. Other studies have supported this finding, demonstrating that nutrition-focused instructions and positive mother-child interactions can enhance mothers’ nutritional practices and improve toddlers’ eating habits [27]. Furthermore, regarding the relationship between children’s developmental state and nutritional behaviors, the findings revealed that children’s developmental state in problem-solving had a statistically significant relationship with nutritional behaviors. The mean food tendency score in children with normal problem-solving skills was significantly higher than those who required a medical referral. Several studies supporting the findings of this study found that nutritional habits and behaviors affect the physical growth and cognitive and emotional development of children [28]. Thus, improving eating behaviors and habits promotes healthy child development [29]. Nutritional support during childhood is crucial for boosting cognitive and functional abilities in childhood and adulthood [30]. As a result, it is essential to prevent developmental disorders in children by empowering mothers, educating them about the value of nutrition in childhood, and raising their awareness of how nutrition affects children’s growth and development.

This study’s mean parent-child relationship score was lower than the tool’s average score. Participants obtained the highest mean score in the conflict dimension and the lowest in the intimacy dimension. Establishing a two-way attachment relationship between the parent and the child results from interaction in the parent-child relationship, which psychologists consider the most crucial factor in child development. Instead of being caused by genetic or biological factors, behavioral disorders in children frequently result from parental communication with them and their methods of upbringing [31], demonstrating the crucial role of parental behavior and parenting techniques in developing behavioral disorders in children and adults. Parents should be encouraged to interact with children and understand their feelings to develop children’s communication skills [32]. In this regard, evidence demonstrates that parent-child interaction improves children’s social skills [7, 33]. Additionally, improving mother-child interaction improves communication and adaptation abilities. On the other hand, a disruption in the child’s development results from parents’ lack of communication skills and insufficient parental support [34].

This study examined the relationship between children’s developmental domains and parent-child interaction. It was found that the communication domain had a significant relationship with the parent-child interaction and its conflict dimension. Children with normal communication had a higher conflict score than those requiring medical referral. Several studies have found that family conflicts and marriage failure, lack of warm family relations, insecure attachment, strict regulations, insufficient supervision, and psychiatric diseases in parents increase the risk of behavioral and emotional problems in children [9]. Furthermore, supporting and communicating with children can effectively reduce aggressive and negative behaviors in toddlerhood while improving the child’s mental health [31, 35]. Also, studies show that responsive and warm parent-child interaction plays an important role in improving the state of development, especially in the aspect of a child’s problem-solving skills [36]. It is also one of the most important effective factors in children’s nutritional behavior. Appropriate mother-child interaction plays an important role in improving child-parent relationships and improving nutritional behaviors [37]. because the first communication between child and parent is formed when the child eats. Mothers who have a better relationship with their children, their children have better behaviors and eating habits [35].

Similar to any other research project, this study faced some limitations. The main limitation of this study was related to the sample size. Therefore, future studies are recommended to investigate the effects of other cultural, social, and psychological factors of families on the growth and development of children in larger samples.

## Conclusion and recommendations

Screening and identifying children at risk as soon as possible is critical for early diagnosis and intervention and reducing future problems. Early diagnosis of developmental disorders in childhood is necessary for preventing chronic diseases and disabilities later in life. Furthermore, the health system managers and practitioners can use the findings of this study and similar studies to develop the necessary plans to monitor the developmental state of children to adopt preventive measures to reduce additional costs imposed on families and society. The main strength of this study is that it provides a broad overview of toddler development and the relationship of nutritional and behavioral factors with developmental dimensions. The study findings revealed that nutritional behavior and parent-child interaction are related to the developmental status of toddlers, an important issue that parents and service providers should consider.

## Data Availability

All data and additional data files in Persian are available from the corresponding author on reasonable request.
